# Iron Supplementation Effects on Redox Status following Aseptic Skeletal Muscle Trauma in Adults and Children

**DOI:** 10.1155/2017/4120421

**Published:** 2017-01-22

**Authors:** Chariklia K. Deli, Ioannis G. Fatouros, Vassilis Paschalis, Athanasios Tsiokanos, Kalliopi Georgakouli, Athanasios Zalavras, Alexandra Avloniti, Yiannis Koutedakis, Athanasios Z. Jamurtas

**Affiliations:** ^1^School of Physical Education and Sport Science, University of Thessaly, Trikala, Greece; ^2^Institute for Research and Technology of Thessaly (I.RE.TE.TH), Trikala, Greece; ^3^Center for Research and Technology Hellas (CERTH), Thessaloniki, Greece; ^4^School of Physical Education and Sport Science, Democritus University of Thrace, Komotini, Greece; ^5^School of Sports, Performing Arts and Leisure, University of Wolverhampton, Wolverhampton, UK

## Abstract

Exercise-induced skeletal muscle microtrauma is characterized by loss of muscle cell integrity, marked aseptic inflammatory response, and oxidative stress. We examined if iron supplementation would alter redox status after eccentric exercise. In a randomized, double blind crossover study, that was conducted in two cycles, healthy adults (*n* = 14) and children (*n* = 11) received daily either 37 mg of elemental iron or placebo for 3 weeks prior to and up to 72 h after an acute eccentric exercise bout. Blood was drawn at baseline, before exercise, and 72 h after exercise for the assessment of iron status, creatine kinase activity (CK), and redox status. Iron supplementation at rest increased iron concentration and transferrin saturation (*p* < 0.01). In adults, CK activity increased at 72 h after exercise, while no changes occurred in children. Iron supplementation increased TBARS at 72 h after exercise in both adults and children; no changes occurred under placebo condition. Eccentric exercise decreased bilirubin concentration at 72 h in all groups. Iron supplementation can alter redox responses after muscle-damaging exercise in both adults and children. This could be of great importance not only for healthy exercising individuals, but also in clinical conditions which are characterized by skeletal muscle injury and inflammation, yet iron supplementation is crucial for maintaining iron homeostasis. This study was registered at Clinicaltrials.gov Identifier: NCT02374619.

## 1. Introduction

Skeletal muscle injury is manifested in several debilitating diseases that are characterized by marked proteolysis and muscle wasting such as cancer [1], muscular dystrophy [2], rheumatoid arthritis [3], and sepsis [4]. Muscle injury causes tissue disruption and subcellular damage followed by cytokine release and a rapid invasion of leukocyte subpopulations into the muscle [5–7]. This inflammatory response is followed by a muscle repair/regeneration phase [5, 8] and muscle recovery [9]. Intense eccentric exercise induces skeletal muscle microtrauma characterized by loss of muscle cell integrity and a marked aseptic inflammatory response evidenced by leukocyte infiltration, edema, hyperthermia, protein release into the plasma, and oxidative stress [4, 6, 10–17], but not sepsis [4]. The remarkable resemblances between exercise-induced aseptic inflammation and muscle trauma make eccentric exercise a valuable tool to investigate muscle inflammation and oxidative stress in humans.

Oxidative stress occurs when the production of free radicals and reactive oxygen and nitrogen species (RONS) overwhelms the antioxidant capacity of the human body. In the injured muscle, RONS are released by inflammatory neutrophils and phagocytes [5, 18]. Additionally, cytokines that are released by neutrophils and the injured myofibers can also lead to increased oxidative stress [19, 20] and further contribute to muscle damage by triggering a secondary sequence of events after the initial trauma [18].

Aseptic inflammation and RONS have been found to facilitate and probably to enhance the restoration of the injured muscle by facilitating the removal of cellular debris [5, 8, 9]. Nevertheless, the typical approach so far was to provide antioxidants to minimize the generation of RONS and restore muscle performance, yet the effectiveness of such an approach is still under debate. In fact, there are studies that report beneficial effects of antioxidants on muscle performance and reduction of oxidative stress [14], whereas others state either no alterations of redox adaptations [12] or even more enhanced oxidative stress due to supplementation [17] and attenuation of the adaptive response of the muscle [21].

Prooxidants are substances characterized as capable of disturbing the redox balance and lead to increased production of RONS that are involved in critical biological processes such as gene expression, signal transduction, and enzyme activity [22–25]. Iron is such a potent prooxidant. Indeed, several pathological conditions are associated with increased oxidative stress due to iron accumulation within cells [26, 27].

Besides its potent prooxidant character, iron is also an essential element for the completion of numerous important biological functions and also for optimal exercise performance. It is a vital component for the formation of oxygen-transport and iron-storage proteins hemoglobin and myoglobin [28] and for the most favorable function of many oxidative enzymes that affect the intracellular metabolism [29]. Therefore, iron supplementation is commonly used to avoid exercise-induced perturbations of iron homeostasis. The effect of iron supplementation on maintaining the required iron stores that are necessary to address exercise needs or enhance physical performance has been studied [30, 31]. In contrast, the prooxidant potential of iron to affect the magnitude of RONS production and alter the usual redox responses following muscle-damaging exercise has not yet been examined. Moreover, despite the extended research conducted in adults, very few studies have addressed the temporal changes in oxidative stress after muscle-damaging exercise in children [32–34]. However, children also experience eccentric muscle contractions in their daily activities (e.g., hopping and jumping) or through their participation in athletic events. Furthermore, children are often subjected to iron supplementation due to iron deficiencies provoked as a result of diet or increased iron needs due to growth [30, 35].

The present study investigated the age-dependent effect of iron supplementation on possible alterations of redox status after muscle-damaging eccentric exercise. Our hypothesis was that iron supplementation would provoke different responses on redox status after muscle-damaging eccentric exercise in an age-dependent manner.

## 2. Subjects and Methods

According to a preliminary power analysis [a probability error of 0.05 and a statistical power of 90% for two groups and two measurements points (pre and post)], a sample size of 10 subjects per group was considered appropriate in order to detect statistically meaningful changes between groups. Power analysis estimations were based on studies that examined the effects of acute exercise on redox biochemical variables (i.e., glutathione, catalase, TAC, PC, TBARS, uric acid, and bilirubin) and CK [13, 36]. In this study 14 healthy recreationally active male adults (18–45 y) and 11 preadolescent males (10–12 y) were enrolled. Both adults and children were participating in several health-promoting physical activities without being systematically trained. Participants' baseline anthropometric characteristics are presented in Table 1. Participation criteria included a normal BMI, normal iron status, absence of lower-limb musculoskeletal injury, no drug/supplement consumption, and absence of any allergy in iron salts. Additionally, they did not undertake any scheduled eccentric exercise or other activities with large eccentric component for at least 6 months before the study, and they did not spend more than 3 h weekly on sport activities. Written informed consent to participate in the study was provided by all adults and children's parents/guardians after they were informed about all risks, discomforts, and benefits involved in the study. Moreover, children's parents approved the participation of their children in the study and provided their written informed consent. Procedures were in accordance with the 1975 Declaration of Helsinki, as revised in 2000, and approval was granted by institutional ethics committee. No funding was received for the completion of the present study.

### 2.1. Supplementation

In a double blind, randomized crossover study that was conducted in two cycles, subjects received daily either the iron supplement [37 mg of elemental iron, Resoferon c.tab 125 (37) mg/tab, Novartis Hellas] or the placebo (lactose) for three weeks prior to and up to 96 h after an acute eccentric exercise bout. The specific dose was selected due to the very novel approach to the scientific problem and due to the fact that it was close to the proposed minimum “therapeutic” requirement that corresponds to 100 mg of iron preparation daily [37]. Additionally, it has been found that supplementation with quantities of 39–50 mg per day of elemental iron for 12 weeks [38] or smaller duration (of even two weeks) [39] adequately improves iron status. Moreover, according to the Food and Nutritional Board, Institute of Medicine [40], the suggested tolerable upper intake level of elemental iron corresponds to 40 mg per day for children aged 9–13 years and to 45 mg per day for individuals aged 14 years and over. A random allocation sequence was applied by the head of the laboratory, using block randomization (block size: two), according to which each individual received the supplement prepacked in daily doses labeled with the day of consumption and was asked to return the package with any capsules left, so that the compliance could be calculated. The supplement was given to the principal investigator by the head of the laboratory upon the arrival of the subjects. Neither the principal investigator, nor the subjects knew whether they were receiving the supplement or the placebo. Subjects were advised, for better absorption, to consume the supplement in the morning on an empty stomach or at least 2 h after breakfast and 2 h before lunch.

### 2.2. Study Design

**Figure 1:**
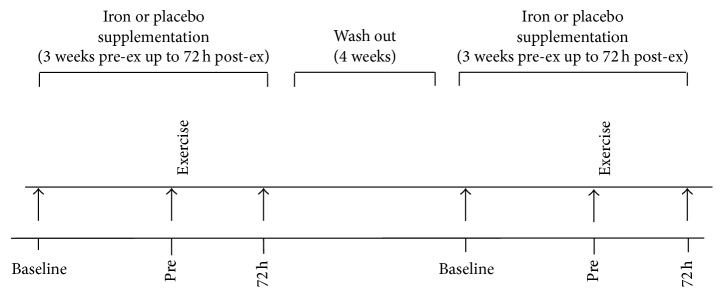
Experimental design of the study. Arrows indicate the time points of creatine kinase and oxidative stress indices assessment.

The experimental approach of the study is presented in Figure 1. During their first visit, subjects' body mass was measured to the nearest 0.05 kg (Beam Balance 710; Seca, Birmingham, United Kingdom) while the subjects were lightly dressed and barefoot. Standing height was measured to the nearest 0.5 cm (Stadiometer 208; Seca). Percentage body fat was calculated from 7 skinfold-thickness measures (average of 2 measurements of each site) by using a Harpenden caliper (John Bull, St Albans, United Kingdom). The Siri skinfold-thickness equation was used to calculate body fat. Biological age of children was assessed by self-estimation of the subjects of Tanner's sexual maturation stages through drawings that depicted the different Tanner stages of sexual maturity and accompanied by the appropriate guidelines, as previously described [41, 42]; the validity of the above method of sexual maturity estimation has been documented [43]. The eccentric exercise protocol was performed on an isokinetic dynamometer (Cybex, Ronkonkoma, NY). Subjects performed the exercise during the first cycle with one leg, after being randomly allocated to one of the two experimental conditions (supplement or placebo) that lasted for 3 weeks prior to and another 96 h after the exercise. Following a 4-week washout period, subjects performed the exercise during the second cycle with the contralateral leg under the second condition. The leg that was used in each cycle was also randomly assigned. Blood samples were collected at baseline, after 2 weeks of supplementation (preexercise), and 72 h following exercise.

### 2.3. Exercise Protocol

A warm-up consisting of 8 min of cycling (80–100 rpm and 50 W) on a Monark cycle ergometer (Monark, Vansbro, Sweden) followed by 5 min of stretching exercises of the major muscle groups of the lower limbs preceded the experimental exercise protocol. Thereafter, participants were placed on the isokinetic dynamometer in a seated position. The preparation of the subject on the dynamometer has been presented in detail elsewhere [44]. The position of each subject was recorded and used in follow-up measurements. At the first cycle, each participant performed 5 sets of 15 maximal eccentric voluntary contractions of knee extensors of their one leg at an angular velocity of 60°·sec^−1^ in the seated position and the same procedures were followed during the second cycle using this time the contralateral leg. A two min rest interval was incorporated between sets. This protocol of eccentric exercise has been used in several studies by our laboratory and is capable of inducing aseptic muscle trauma and oxidative stress [12, 13, 45].

### 2.4. Blood Collection and Handling

Blood samples (10 mL) were drawn from a forearm vein and plasma and red blood cells lysate were collected as previously described [46]. A blood aliquot (1.5 mL) was collected into ethylenediaminetetraacetic acid (EDTA) tubes for the estimation of hematological parameters. For serum, blood was collected into tubes containing coagulation agent and after staying for 20 min to clot, it was centrifuged at 1370 ×g for 10 min at 4°C, and serum was collected. Plasma, erythrocytes lysate, and serum were stored in multiple aliquots at −80°C and thawed only once before the analyses.

### 2.5. Assays

#### 2.5.1. Iron Status

Iron concentration and total iron binding capacity (TIBC) were estimated in a Clinical Chemistry Analyzer Z 1145 (Zafiropoulos Diagnostica, Athens, Greece) with commercially available kits (Zafiropoulos, Athens, Greece). Each sample was analyzed in duplicate. Transferrin saturation (TS) was calculated through the ratio of iron concentration and TIBC (TS = iron concentration/TIBC × 100). For the determination of serum ferritin, an immunoenzymometric assay kit based on sandwich ELISA was used (Accubind®, Monobind Inc., USA). Inter- and intra-assay coefficients of variation (CV) for all blood parameters ranged from 2.1 to 6.5% and from 2.9 to 7.1%, respectively.

#### 2.5.2. Muscle Damage

Serum creatine kinase (CK) was measured in a Clinical Chemistry Analyzer Z 1145 (Zafiropoulos Diagnostica, Athens, Greece) with commercially available kits (Zafiropoulos, Athens, Greece). The intra- and inter-assay CV for CK were 3.9% and 5.9%, respectively.

#### 2.5.3. Redox Status

Thiobarbituric reactive substances (TBARS), protein carbonyls (PC), reduced glutathione (GSH), catalase (CAT), and total antioxidant capacity (TAC) were determined in a HITACHI, U-1900 spectrophotometer as previously described [46]. The intra- and inter-assay CV for TBARS were 3.9% and 5.9%, for protein carbonyls 4.3% and 7.0%, for GSH 3.1% and 4.5%, for catalase 6.2% and 10.0%, and for TAC 2.9% and 5.4%, respectively.

Uric acid and bilirubin were estimated in a Clinical Chemistry Analyzer Z 1145 (Zafiropoulos Diagnostica, Athens, Greece) with commercially available kits (Zafiropoulos, Athens, Greece). Each sample was analyzed in duplicate. The intra- and inter-assay CV for uric acid and bilirubin were 4.5% and 3.4%, respectively.

### 2.6. Dietary Analysis

The subjects were instructed to follow their usual eating habits during the days before the experiment and were also asked to record their diet 3 days prior to exercise bouts as previously described [47, 48]. Dietary records were analyzed with ScienceFit Diet 200A (Science Technologies, Athens, Greece). The subjects received a copy of their dietary record sheets and were asked to follow exactly the same food intake patterns (as recorded in their dietary record sheets) before their second experimental session.

### 2.7. Statistical Analysis

The normality of the sample distribution was examined with a Kolmogorov-Smirnov test. Ferritin, TBARS, TAC, and CK were subjected to logarithmic transformation as previously described [49]; after transformation skewed distributions tended towards symmetry. Differences in anthropometric characteristics at baseline, between adults and children, were estimated through unpaired Student's *t*-test.

To estimate possible differences as a result of 3 weeks of iron supplementation in iron status, and redox status in adults compared to children, a three-way ANOVA [time (baseline-3 wks) × condition (placebo-iron supplement) × age (adults-children)] with repeated measures on time was used.

To assess possible differences in redox status after eccentric exercise as a result of iron supplementation in adults compared to children, a three-way ANOVA [time (pre-72 h) × condition (placebo-iron supplement) × age (adults-children)] with repeated measures on time was performed.

When significant main effects or interactions occurred, Sidak pairwise comparisons were applied. The level of statistical significance was set at *p* < 0.05. For all statistical analyses SPSS, version PASW 18.0 (SPSS Inc., Chicago, IL) was used. The results are presented as M ± SEM.

## 3. Results

### 3.1. Anthropometric Characteristics and Dietary Intake

Anthropometric characteristics are presented in Table 1. Adults demonstrated higher values compared to children.

Dietary intake is presented in Table 2. No significant differences were found in daily energy and macronutrient intakes between adults and children.

### 3.2. Iron Supplementation Effects at Rest

#### 3.2.1. Iron Status

Main effect of time existed for FE concentration (*p* < 0.05), while time × condition interaction existed for FE concentration (*p* < 0.001) and TS (*p* < 0.01). Iron supplementation for 3 weeks increased FE concentration (mg/dL) and TS (%) by 28% in both age groups (*p* < 0.001). Iron supplementation did not change TIBC or ferritin. Detailed iron status changes from baseline after 3 weeks are presented in Table 3.

Main effect of age existed for FE concentration (*p* < 0.05), TS (*p* < 0.01), TIBC (*p* < 0.05), and ferritin (*p* < 0.001). Adults had higher FE, TS, and FERR (*p* < 0.001) but lower TIBC compared to children (Table 3).

#### 3.2.2. Blood Redox Status

Main effect of time existed for TBARS (*p* < 0.01). TBARS similarly increased after 3 weeks in both the iron and placebo condition (Table 4).

Main effect of age existed for bilirubin (*p* < 0.01), uric acid (*p* < 0.001), CAT (*p* < 0.01), and GSH (*p* < 0.01). Children had higher GSH and CAT but lower uric acid and bilirubin compared to adults (Table 4).

#### 3.2.3. Muscle Damage

No changes occurred in CK activity in all groups.

### 3.3. Iron Supplementation Effects after Eccentric Exercise

#### 3.3.1. Muscle Damage

**Figure 2:**
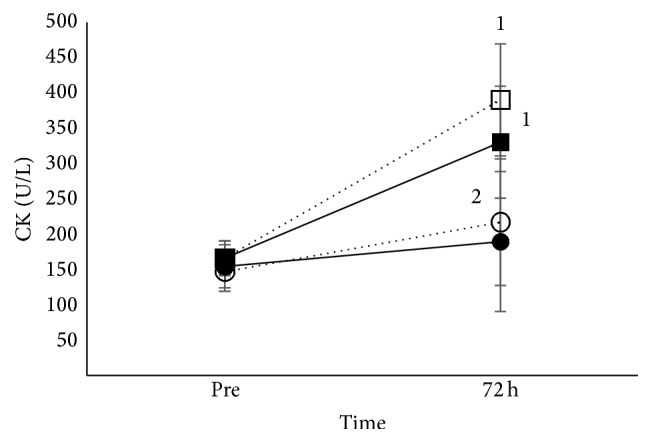
CK after eccentric exercise in adults and children. Mean (±SEM) creatine kinase activity (CK), in adults under iron (■) and placebo supplementation (□) and in children under iron (●) and placebo supplementation (○). ^1^Different from preexercise in the same group. ^2^Different between adults and children at the same time point.

Main effect of age (*p* < 0.05), main effect of time (*p* < 0.001), and time × age interaction (*p* < 0.01) existed for CK activity. Adults' CK activity increased at 72 h after eccentric exercise in both iron and placebo condition, while no changes occurred in children (Figure 2). Additionally, adults presented higher CK activity compared to children in the whole study (Figure 2).

#### 3.3.2. Blood Redox Status

**Figure 3:**
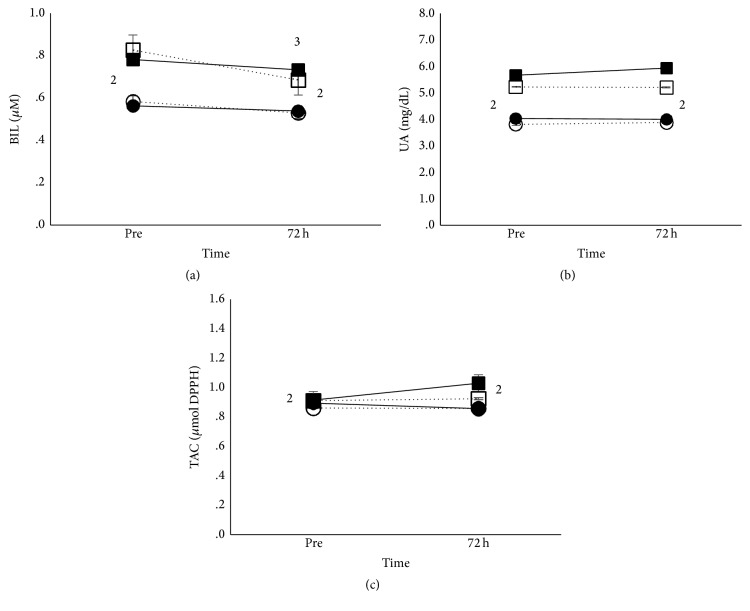
Plasma antioxidants after eccentric exercise in adults and children. Mean (±SEM) bilirubin (a), uric acid (b), and TAC (c) in adults under iron (■) and placebo supplementation (□) and in children under iron (●) and placebo supplementation (○). ^2^Different between adults and children at the same time point. ^3^Different from preexercise in all groups.

Main effect of time existed for bilirubin (*p* < 0.5) and TBARS (*p* < 0.01), while time × condition interaction (*p* < 0.05) existed for TBARS. Bilirubin decreased at 72 h after eccentric exercise in all groups (Figure 3). Iron supplementation increased TBARS at 72 h after eccentric exercise in both age groups, while no changes were observed under placebo supplementation (Figure 5). No changes occurred in PC after eccentric exercise in all groups.

**Figure 4:**
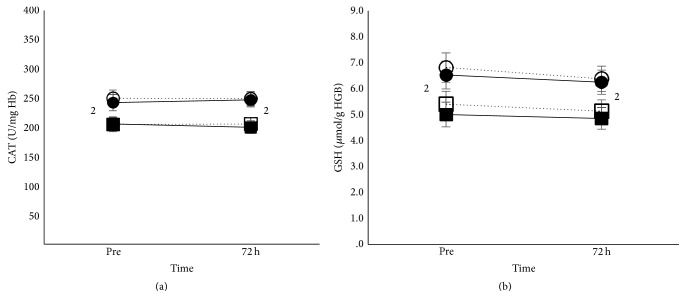
Erythrocytes' antioxidants after eccentric exercise in adults and children. Mean (±SEM) catalase (a) and GSH (b), in adults under iron (■) and placebo supplementation (□) and in children under iron (●) and placebo supplementation (○). ^2^Different between adults and children at the same time point.

**Figure 5:**
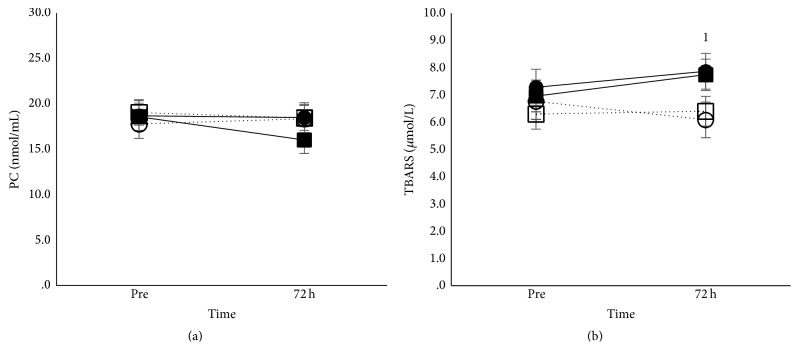
Protein and lipid oxidation after eccentric exercise in adults and children. Mean (±SEM) protein carbonyls (PC) (a) and TBARS (b), in adults under iron (■) and placebo supplementation (□) and in children under iron (●) and placebo supplementation (○). ^1^Different from preexercise in iron condition.

Main effect of age existed for bilirubin (*p* < 0.01), uric acid (*p* < 0.001), catalase (*p* < 0.01), GSH (*p* < 0.01), and TAC (*p* < 0.05). Adults had higher bilirubin, uric acid, and TAC (Figure 3) but lower catalase and GHS (Figure 4) compared to children in the whole study, independently of condition.

## 4. Discussion

The present study investigated the effects of iron supplementation, on redox status after aseptic muscle trauma induced by eccentric exercise. To our knowledge, this is the first attempt to investigate the effects of a potent prooxidant on blood redox status in adults and children after eccentric exercise. The results of the present study indicate that iron supplementation results in greater lipid peroxidation following eccentric exercise and this effect seems not to be age related.

### 4.1. Muscle Damage

Creatine kinase is a muscle-derived protein and under normal situations its concentration in the blood is low. When muscle trauma occurs, as it happens after muscle-damaging exercise, there is an increased flux of CK outside of the muscle, and as a result its activity in the blood also increases [50]. The increase of CK activity after exercise-induced muscle trauma has been reported to be as high as 150-fold the preexercise levels [19], usually peaking between 2 and 5 days [13, 50–53], while lasting up to 7 days after the cessation of exercise [49]. In the present study, the elevation of CK activity into the circulation 72 h following eccentric exercise in adults indicates that muscle damage was provoked. Interestingly, CK activity was not elevated at 72 h in children. However, this does not necessarily mean that no changes in CK activity occurred in children. In the present study, for reasons explained in the methods section, blood drawings were performed only twice, that is, before and at 72 h after eccentric exercise. However, children's CK activity could have been peaked at a preceding time point, as previously has been reported [33, 54], and had already been recovered by 72 h.

### 4.2. Redox Status

Increased generation of RONS for several days after the termination of eccentric [6, 12–14, 16, 55] or eccentrically biased exercise [11, 56] has been reported by a great number of studies. In adults, increased oxidation of human biomolecules usually occurs after the first 24 h lasting up to 8 days [12–14, 16, 17, 55], although increased protein oxidation 2 h [6] and lipid peroxidation 6 h [55, 56] after exercise have also been reported. The concentration of blood antioxidants also increases at several time points after muscle-damaging exercise except for GSH that usually decreases, with the time-course of changes ranging between some hours [6, 14, 55] and days after exercise [12, 13, 17]. Contrary with adults, data regarding exercise-induced oxidative stress in children [45, 57] and adolescents [58] is scarce. This data provides some evidence of oxidative stress being induced also in children after exercise, yet no further assessment of redox indices has been performed the following days.

In this study, iron supplementation altered redox status responses after muscle-damaging exercise, providing evidence in favor of its prooxidant character. TBARS increased only under iron supplementation in both age groups, while bilirubin decreased in all groups as a result of muscle-damaging exercise. However, similarly with CK, the absence of any redox status index alteration at 72 h under placebo condition does not mean that a modulation of redox status has not occurred at an earlier time point than that of 72 h. The absence of any blood redox status assessment between preexercise and 72 h following exercise may have shielded the true redox responses. This means that redox status perturbations may have happened at an earlier time point, as shown in previous studies [6, 12–14, 16, 55]. Nevertheless, even if redox status perturbations have happened earlier in the study, the absence of redox status changes at 72 h under placebo condition denotes attenuated redox status changes and faster recovery under placebo, compared to iron condition. Except for increased production of free radicals, eccentric exercise-induced muscle trauma leads also to aseptic inflammation, evidenced by changes in several proinflammatory cytokines (IL-6, IL-I*β*, and TNF*α*) [6, 9, 10, 17] or anti-inflammatory cytokines (IL-10, IL-12, and IL-1ra) [6, 9, 10]. The present study did not assess such cytokines, or ROS levels, and this constitutes a limitation of the study. Future research should incorporate the assessment of pro- and anti-inflammatory cytokines for a more spherical depiction of the exercise-induced aseptic muscle trauma.

The oxidative damage from iron comes mainly from the interaction of Fe^2+^ with H_2_O_2_ to produce hydroxyl radical (HO^•^) through Fenton reaction. HO^•^ can rapidly react with most molecules, including lipids and proteins [59]. In situations of exercise-induced muscle trauma, Fenton reaction can be triggered by neutrophils and macrophages gathered in the injured area due to phagocytosis and the subsequent “oxidative burst” [60]. Additionally, the destruction of hemoglobin and myoglobin due to hemolysis that occurs after muscle-damaging eccentric exercise [12] can also contribute to increased production of free radicals by releasing catalytic iron from erythrocytes [61]. The increased amounts of iron due to supplementation prior to exercise in the present study may have led to increased lipid peroxidation (as indicated by the increase in TBARS) through the above mechanisms after eccentric exercise.

Iron-mediated alteration of redox environment could also have an impact on* HMOX-1*, the gene coding for HMOX1 enzyme, that is specific to iron metabolism [62]. The activation of HMOX1 is involved in heme catabolism and is induced by oxidative stress. HMOX1 catalyzes the degradation of heme to biliverdin (which is subsequently converted to bilirubin by biliverdin reductase), carbon monoxide, and Fe^2+^ [63]. The released Fe^2+^ in turn drives the synthesis of ferritin, which acts as a protectant against oxidant damage [62]. This mechanism has been considered as a heme oxygenase-induced defense against oxidative stress, because iron is stored in ferritin in an inactive form [64, 65]. The present study did not examine HMOX1 activity and ferritin concentration after eccentric exercise (and this constitutes a second limitation), and therefore it cannot provide evidence in favor of an alteration of iron homeostasis due to the above mechanism. However, we have assessed the concentration of bilirubin, which was found to be decreased at 72 h after the induction of exercise-induced aseptic muscle trauma. This decrement of bilirubin does not necessarily indicate that HMOX1 activity was not altered after eccentric exercise. The lack of bilirubin assessment at a previous time point compared to that of 72 h may have shielded an earlier increase of bilirubin and consequently an earlier increase in HMOX1 activity. Therefore, the low levels of bilirubin at 72 h after compared to before exercise may indicate its extended use the previous days to combat the increased lipid peroxidation provoked by eccentric exercise.

**Figure 6:**
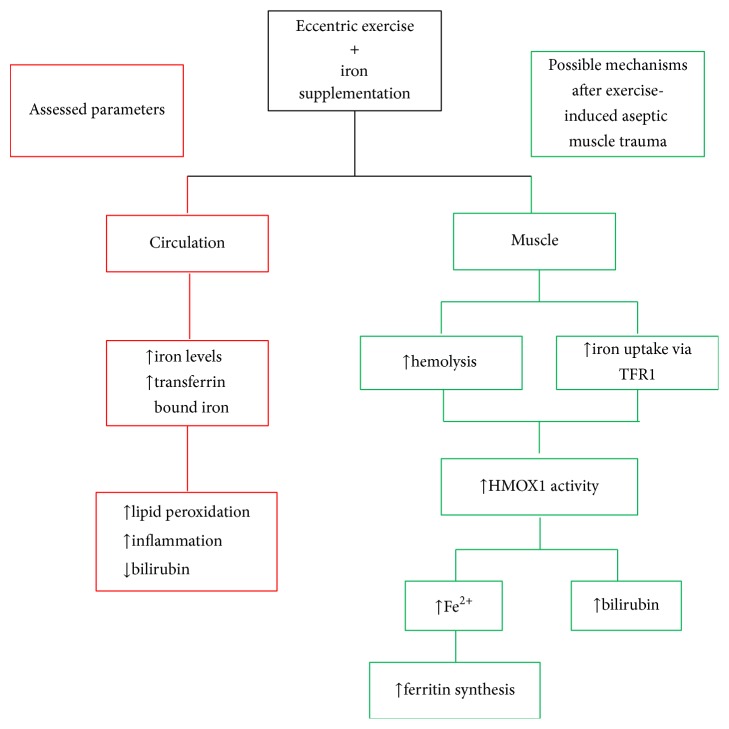
Study results and possible mechanisms triggered after iron-mediated exercise-induced aseptic muscle trauma.

Iron supplementation could also affect iron transporters both prior to and after exercise-induced aseptic muscle trauma. Dietary iron is exported from the enterocytes into the circulation and its transport is mediated by transferrin, which limits the ability of iron to generate toxic radicals, thereby protecting the organ systems from the toxic effects of iron [66]. Transferrin also facilitates the delivery of transferrin-bound iron to cells through the pathway of transferrin receptor 1 (TFR1) [64]. The binding of transferrin to TFR1 causes the protein complex to be taken into the cell by receptor-mediated endocytosis. Once iron is released, it is then transported across the endosomal membrane and into the cell by divalent metal-ion transporter 1 (DMT1) [64]. The amount of transferrin-bound iron taken up by the cells is regulated by the binding of iron regulatory proteins (IRPs) to specific RNA stem-loop structures called iron-responsive elements (IREs) [67]. Iron deprivation strongly induces the RNA binding activity of IRPs to IREs; binding of IRPs to the 3 UTR of the TFR1 mRNA results in an increase in the expression of TFR1, while IRP binding to the single IRE situated in the 5 UTR of ferritin mRNAs prevents translation of the ferritin protein. In contrast, when the cell is iron replete, IRP binding to IRPs decreases, reducing that way TFR1 protein levels and consequently iron uptake, while simultaneously allowing the translation of ferritin to safely store excess iron [67]. The latter scenario is more likely to be true in the present study. Iron supplementation-induced oxidative stress after eccentric exercise in conjunction with the hemolysis that occurs after eccentric exercise [12] may have led to the induction of HMOX1 and increased concentration of Fe^2+^ into the muscle, thereby stimulating ferritin synthesis. Unfortunately, such information is not available in the present study, due to lack of ferritin assessment. Therefore, future research should incorporate the evaluation of ferritin, but also TFR1, to clarify if iron-mediated oxidative stress could also affect iron transportation in situations of exercise-induced aseptic muscle trauma. The above possible mechanisms triggered by iron-mediated increase of oxidative stress after exercise-induced aseptic muscle trauma are presented in Figure 6.

Except for the increase in TBARS and the decline in bilirubin concentration, no other redox status marker was significantly modified in the present study. The reason for that could be the specific low to moderate dose or the rather short duration of supplementation, which, although being capable of increasing TBARS production, is not sufficient to affect protein oxidation or other endogenous antioxidants. Therefore, a higher dose, or a longer supplementation duration, could probably lead to different results. Nevertheless, the present response of TBARS and bilirubin after iron supplementation denotes the possibility of iron to differently modify redox responses and adversely affect aseptic muscle trauma.

Iron's capability of altering redox environment could be of great importance not only for healthy exercising individuals, but also in clinical conditions which are characterized by skeletal muscle injury and inflammation, and on the other hand, iron supplementation is crucial for maintaining iron homeostasis [68]. Malondialdehyde, one of the main end products of lipid peroxidation (which in the present study is quantified through its reaction with TBA), can react with DNA bases guanine, adenine, and cytosine to form M_1_G, M_1_A, and M_1_C adducts, respectively [69]. These oxidized DNA products are mutagenic and carcinogenic and represent a good biomarker of oxidative stress of an organism and carcinogenesis [70]. Indeed, M_1_G adducts were detected to be significantly elevated in human breast tissues and rodent tissues [70]. Except for cancer, MDA is also associated with Alzheimer's disease [24], rheumatoid arthritis [71], atherosclerosis, and diabetes [24].

In the present study, no assessment of redox status beyond 72 h was made, and no information can be given regarding the duration of and recovery from iron-mediated lipid peroxidation after eccentric exercise. Therefore, future research should incorporate a longer postexercise period of oxidative stress assessment, for better understanding of the magnitude of iron-mediated redox changes in exercise-induced aseptic muscle trauma.

In the present study, iron supplementation similarly modulated oxidative stress indices in both adults and children. Despite the proposed greater ROS release in children and adolescents in response to exercise than in adults due to their faster VO_2_ kinetics and the observed robust increases in circulating neutrophils in children after exercise [72], this was not the case in the present study. The attenuated CK activity in children compared to adults could lead to the assumption that the extent of muscle damage provoked in children would be lower compared to adults. This could have also resulted in lower inflammation-mediated ROS production and lower hemoglobin and myoglobin catabolism [73], consequently leading to attenuated oxidative stress in children compared to adults. However, in the present study redox status responses in children were similar with the ones of adults. As has already been mentioned, there is limited data in children [45, 57] and adolescents [58] regarding exercise-induced redox alterations and no data comparing those alterations between adults and children. Submaximal exercise on a stationary cycle ergometer at 70% VO_2max_ increased TBARS, PCs, GSSG, TAC, CAT, and GPX but decreased GSH and the ratio of GSH : GSSG in prepubertal and early pubertal male children [57]. Similarly, Nikolaidis et al. [45] reported significant increases in TBARS, PC, CAT, TAC, and oxidized glutathione (GSSG) concentration, as well as significant decreases in GSH and GSH : GSSG, after 12 bouts of 50 m swimming, performed at 70%–75% of 50 m maximum velocity. The results from a more recent study from our laboratory [58] indicate a greater rise in PC, TBARS, and TAC and greater GSH decline in adults compared to adolescents following an aerobic exercise bout. Uric acid, CAT, and bilirubin were similar amongst adults and adolescents immediately after and 1 h following the aerobic trial [58]. Although no comparison between children and adults was made in the aforementioned studies, similarly to our study, their results also show that oxidative stress occurs in youngsters. The fact that more redox indices were modified in those studies compared to our study could be due to the greater aerobic component of the exercise protocols used in those studies and the different time points of assessment.

The results of the present study point out the critical role of age on endogenous antioxidants regardless of supplementation. Children's erythrocytes presented greater antioxidant capacity compared to adults as denoted by the higher GSH and CAT activity, while the opposite was observed for plasma antioxidants with adults presenting higher uric acid, bilirubin, and TAC. The higher GSH and CAT activity in children could be due to the lower overall destruction of senescent erythrocytes by the reticuloendothelial system and a lower overall iron turnover [74]. If the number of erythrocytes removed daily by macrophages in children is less compared to adults, then functional iron released from catabolized hemoglobin [59, 75, 76] may also be less; this probably results in reduced production of ROS and lower need for the use of GSH and CAT in children. The higher uric acid and bilirubin concentrations in adults could be explained as a protective response to increased phagocytosis of senescent erythrocytes and the higher ROS generation [77, 78] compared with children. Additionally, the higher uric acid in adults in the present study could also be due to increased intake of purine-rich foods [77, 79, 80], older age, higher BMI, greater height, and greater weight compared to children [79]. The differences in antioxidant status between adults and children of the present study were apparent at rest, but also during the recovery period after eccentric exercise. Nevertheless, the produced oxidative stress was similar between the two age groups. Although TAC was greater in adults compared to children following eccentric exercise, lipid peroxidation was similar. It seems that the greater erythrocytes' antioxidant capacity of children compensates for the lower plasma's antioxidant capacity compared to adults, leading that way to a similar redox response to muscle damage. However, more research is needed before any conclusions are drawn regarding the differences in antioxidant status between adults and children and the possible mechanisms underlying these differences.

## 5. Conclusions

This was the first attempt to determine whether iron, a potential prooxidant, could differently modify muscle damage symptoms and redox status after muscle-damaging exercise in adults and children. Iron supplementation, as prescribed in the present study, managed to differently affect redox status, by inducing greater oxidative stress compared to placebo condition after exercise-induced aseptic muscle trauma. Furthermore, the results of the present study support the so far limited data indicating that oxidative stress also occurs in children as in adults, yet more research is warranted in order to verify this resemblance.

The present study investigated the effect of chronic iron supplementation prior to eccentric exercise. Acute effect of iron supplementation starting immediately after exercise-induced muscle damage was not examined, and future studies should address this issue. Additionally, other exercise stimuli, redox status indices, and inflammation markers should also be incorporated in future research. Furthermore, redox status should be assessed for a longer period, at least up to 8 days following exercise-induced aseptic muscle trauma, for better depiction of the time-course of changes in children compared to adults. Finally, due to the majority of the research focusing on males, all of the above issues should be addressed also in women and girls, to determine possible gender-responsible differences.

## Figures and Tables

**Table 1:** Anthropometric characteristics of the participants at baseline.

Anthropometry	Adults (*n* = 14)	Children (*n* = 11)
Age (y)	34.9 ± 2.3^2^	11 ± 0.2
Height (cm)	179 ± 2.2^2^	154 ± 1.5
Weight (kg)	78.0 ± 2.5^2^	42.3 ± 2.1
BMI (kg/m^2^)	24.2 ± 0.6^2^	17.9 ± 0.7
Body fat (%)	19.1 ± 1.8^2^	7.6 ± 1.2
Tanner stage	—	2.4 ± 0.15

All values are means ± SEMs. ^2^Significantly different between adults and children.

**Table 2:** Analysis of daily energy intake in adults and children at baseline.

	Adults (*n* = 14)	Children (*n* = 11)
Energy (kcal)	1940 ± 133.3	1857 ± 120.3
Carbohydrate (% of energy)	51.4 ± 2.3	56.3 ± 1.6
Fat (% of energy)	25.7 ± 1.7	23.3 ± 1.2
Protein (% of energy)	22.9 ± 1.5	20.4 ± 1.0
Iron (mg)	12.1 ± 1.1 (120% RDA)^↑^	10.7 ± 0.7 (100% RDA)^↑^
Vitamin C (mg)	133.6 ± 29.0 (148% RDA)^↑^	87.1 ± 13.4 (193% RDA)^↑^

All values are means ± SEMs. RDA, Recommended Daily Allowance; ^↑^Trumbo et al. [40].

**Table 3:** Iron status after 3 weeks of iron supplementation in adults and children at rest.

Iron status indices	Adults				Children			
	Iron supplementation		Placebo supplementation		Iron supplementation		Placebo supplementation	
	Baseline	3 weeks	Baseline	3 weeks	Baseline	3 weeks	Baseline	3 weeks
FE (mg/dL)	89 ± 8.0^2^	118 ± 8.7^1,2^	100 ± 8.0^2^	77 ± 8.7^2^	58 ± 9.0	90 ± 9.7^1^	79 ± 9.0	86 ± 9.7
TIBC (*μ*mol/L)	387 ± 22.0^2^	396 ± 20.6^2^	380 ± 22.0^2^	353 ± 20.6^2^	433 ± 24.9	403 ± 23.2	421 ± 24.9	431 ± 23.2
TS (%)	24 ± 2.5^2^	31 ± 2.5^1,2^	28 ± 2.5^2^	22 ± 2.5^2^	15 ± 3.0	23 ± 3.0^1^	20 ± 2.8	20 ± 2.8
FERR (g/mL)	94 ± 16.3^2^	95 ± 16.3^2^	95 ± 16.3^2^	92 ± 16.3^2^	20 ± 19.3	21 ± 19.3	25 ± 18.4	23 ± 18.4

All values are means ± SEM. FE, iron concentration; TIBC, total iron binding capacity; TS, transferrin saturation; FERR, ferritin. ^1^Different from baseline in the same group. ^2^Different between adults and children at the same time point.

**Table 4:** Blood redox status in adults and children after 3 weeks of supplementation, at rest.

Redox status indices	Adults				Children			
	Iron supplementation		Placebo supplementation		Iron supplementation		Placebo supplementation	
	Baseline	3 weeks	Baseline	3 weeks	Baseline	3 weeks	Baseline	3 weeks
GSH (*μ*mol/g HGB)	5.3 ± 0.48^2^	5.5 ± 0.47^2^	5.2 ± 0.48^2^	6.0 ± 0.47^2^	6.4 ± 0.57	6.8 ± 0.56	6.6 ± 0.54	6.5 ± 0.53
CAT (U/mg HGB)	200 ± 11.2^2^	205 ± 12.3^2^	202 ± 11.2^2^	206 ± 12.3^2^	235 ± 12.7	250 ± 13.8	238 ± 12.6	242 ± 13.8
TAC (*μ*mol DPPH)	0.92 ± 0.02	0.92 ± 0.02	0.88 ± 0.02	0.91 ± 0.02	0.88 ± 0.02	0.88 ± 0.02	0.86 ± 0.02	0.86 ± 0.02
UA (mg/dL)	5.7 ± 0.36^2^	0.7 ± 0.36^2^	5.5 ± 0.36^2^	0.4 ± 0.37^2^	3.7 ± 0.41	4.0 ± 0.41	3.8 ± 0.41	3.8 ± 0.41
BIL (*μ*M)	0.81 ± 0.08^2^	0.78 ± 0.07^2^	0.73 ± 0.08^2^	0.83 ± 0.07^2^	0.55 ± 0.09	0.55 ± 0.08	0.55 ± 0.09	0.58 ± 0.08
TBARS (*μ*mol/L)	6.5 ± 0.58	6.8 ± 0.59^1^	6.1 ± 0.56	6.3 ± 0.56^1^	6.4 ± 0.63	7.0 ± 0.63^1^	6.5 ± 0.66	6.8 ± 0.66^1^
PC (nmol/mL)	17.7 ± 1.29	18.4 ± 1.43	20.7 ± 1.24	19.0 ± 1.37	17.8 ± 1.40	18.6 ± 1.55	20.3 ± 1.4	17.8 ± 1.55

All values are M ± SEMs. GSH, reduced glutathione; CAT, catalase; TAC, total antioxidant capacity; UA, uric acid; BIL, bilirubin; TBARS, thiobarbituric reactive substances; PC, protein carbonyls. ^1^Significantly different from baseline in the same group. ^2^Significantly different between adults and children independently of condition or time.
